# Grape Proanthocyanidin Inhibit Pancreatic Cancer Cell Growth *In Vitro* and *In Vivo* through Induction of Apoptosis and by Targeting the PI3K/Akt Pathway

**DOI:** 10.1371/journal.pone.0043064

**Published:** 2012-08-08

**Authors:** Ram Prasad, Mudit Vaid, Santosh K. Katiyar

**Affiliations:** 1 Birmingham Veterans Affairs Medical Center, Birmingham, Alabama, United States of America; 2 Department of Dermatology, University of Alabama at Birmingham, Birmingham, Alabama, United States of America; 3 Nutrition Obesity Research Center, University of Alabama at Birmingham, Birmingham, Alabama, United States of America; 4 Comprehensive Cancer Center, University of Alabama at Birmingham, Birmingham, Alabama, United States of America; Vanderbilt University Medical Center, United States of America

## Abstract

Pancreatic cancer is an aggressive malignancy that is frequently diagnosed at an advanced stage with poor prognosis. Here, we report the chemotherapeutic effects of bioactive proanthocyanidins from grape seeds (GSPs) as assessed using *In Vitro* and *In Vivo* models. Treatment of human pancreatic cancer cells (Miapaca-2, PANC-1 and AsPC-1) with GSPs *In Vitro* reduced cell viability and increased G2/M phase arrest of the cell cycle leading to induction of apoptosis in a dose- and time-dependent manner. The GSPs-induced apoptosis of pancreatic cancer cells was associated with a decrease in the levels of Bcl-2 and Bcl-xl and an increase in the levels of Bax and activated caspase-3. Treatment of Miapaca-2 and PANC-1 cells with GSPs also decreased the levels of phosphatidylinositol-3-kinase (PI3K) and phosphorylation of Akt at ser^473^. siRNA knockdown of PI3K from pancreatic cancer cells also reduced the phosphorylation of Akt. Further, dietary administration of GSPs (0.5%, w/w) as a supplemented AIN76A control diet significantly inhibited the growth of Miapaca-2 pancreatic tumor xenografts grown subcutaneously in athymic nude mice, which was associated with: (*i*) inhibition of cell proliferation, (*ii*) induction of apoptosis of tumor cells, (*iii*) increased expression of Bax, reduced expression of anti-apoptotic proteins and activation of caspase-3-positive cells, and (*iv*) decreased expression of PI3K and p-Akt in tumor xenograft tissues. Together, these results suggest that GSPs may have a potential chemotherapeutic effect on pancreatic cancer cell growth.

## Introduction

Pancreatic cancer is considered to be the fourth leading cause of cancer-related deaths in the United States. It is an aggressive malignancy that is frequently diagnosed at an advanced stage with poor prognosis; the overall 5-year survival rate is <5% [1], [2]. Only 20% of pancreatic cancer patients are eligible for surgical resection, which remains the only potential curative therapy [3]. Although a combination of chemotherapy and radiation therapy can improve survival, most patients die of disease progression that often is associated with acquired or intrinsic resistance to chemotherapy [4], [5]. Therefore, the development of more effective therapeutic strategies that can target the molecules associated with pancreatic tumor growth and circumvent or overcome resistance to apoptosis may lead to improved outcomes in patients suffering from pancreatic cancer.

Non-toxic phytochemicals offer promising options for the development of effective chemotherapeutic strategies for various types of cancer. Some case-controlled epidemiologic studies support the concept that the consumption of bioactive dietary phytochemicals reduces the risk of pancreatic cancer [6], [7]. Grape seed proanthocyanidins (GSPs) are bioactive phytochemicals that have shown anti-carcinogenic activity in some animal tumor models [8], http://carcin.oxfordjournals.org/cgi/content/full/24/8/1379 – B10#B10and appear to exhibit minimal toxicity in laboratory animals [9], [10]. GSPs are readily extracted from grape-seeds, and are a mixture of dimers, trimers, tetramers, and oligomers of monomeric catechins and/or (−)-epicatechins [9], [10]. GSPs have been shown to have anti-inflammatory, anti-oxidant and anti-metastatic properties in both *in vitro* and *in vivo* models [8]–[12]. They inhibit ultraviolet radiation- and chemical carcinogen-induced skin carcinogenesis in mouse models [9], [13]. Recently, we have shown that dietary administration of GSPs with AIN76A control diet resulted in a dose-dependent inhibition of the growth of non-small cell lung cancer tumor xenografts [14]. GSPs inhibit the invasive potential of melanoma and head and neck squamous cell carcinoma cells as assessed using *in vitro* models [11], [12]. However, the anti-carcinogenic potential of GSPs against pancreatic cancer is largely unexplored.

The phosphatidylinositol 3′-kinase (PI3K)/Akt pathway is a fundamental signaling pathway that mediates several cellular processes, including cell proliferation, growth, cell survival and motility [15]. Increased activation and deregulation of the components in the PI3K/Akt pathway have been implicated in many malignancies and in conferring resistance to chemotherapy [16]. Akt is a well characterized serine/threonine kinase. Increased Akt activity has been implicated in several types of cancers, where it promotes cell survival through effects on numerous downstream targets, including the inactivation of pro-apoptotic proteins, activation of anti-apoptotic genes and the progression of the cell cycle [17]. Activation of Akt is a frequent event in pancreatic cancer and is associated with poor prognostic variables and outcomes [18]–[20]. One study revealed that 59% of pancreatic adenocarcinomas showed hyperactivation of Akt [18]. Some studies indicate that inhibition of the PI3K/Akt pathway sensitizes pancreatic cancer cells to the apoptotic effect of chemotherapy both *in vitro* and *in vivo* [21]–[23].

In the present study, we assessed the chemotherapeutic effect of GSPs on pancreatic cancer using the Miapaca-2, PANC-1 and AsPC-1 human pancreatic cancer cell lines as *in vitro* cell culture models. To verify the *in vitro* data, we conducted *in vivo* tumor xenograft studies. Our results show that treatment of pancreatic cancer cells with GSPs results in inhibition of cell proliferation, induction of apoptosis and inhibition of the PI3K/Akt pathway.

## Materials and Methods

### Antibodies and reagents

The primary antibodies were obtained as follows: antibodies specific for Bax, Bcl-2, Bcl-xl, cleaved caspase-3, PI3K, Akt, p-Akt and β-actin were purchased from Cell Signaling Technology (Beverly, MA); Cyclin B1, Cdc25B, Cdc25c, Ki-67, and the secondary antibodies, which were horseradishperoxidase-conjugated, were purchased from Santa Cruz Biotechnology, Inc. (Santa Cruz, CA). The PI3K siRNA kit was procured from Santa Cruz Biotechnology, Inc. (Santa Cruz, CA). The Annexin V-conjugated AlexaFluor488 Apoptosis Detection Kit was purchased from Molecular Probes, Inc. (Eugene, OR). The protein assay kit was from Bio-Rad (Hercules, CA). MTT (3-[4,5-dimethyl-2-yl]-2,5-diphenyl tetrazolium bromide) and all other chemicals were of analytical grade and purchased from Sigma Chemical Co. (St. Louis, MO). The GSPs were obtained from the Kikkoman Corporation (Japan). The chemical composition and stability of GSPs have been described previously [9], [10].

### Cells and culture conditions

Human pancreatic cancer cell lines, AsPC-1, PANC-1 and Miapaca-2, were obtained from American Type Culture Collection (Rockville, MD), and cultured as recommended as monolayers in DMEM supplemented with 10% heat-inactivated fetal bovine serum (Hyclone, Logan, UT), 100 µg/mL penicillin and 100 µg/mL streptomycin from Invitrogen (Carlsbad, CA) in a humidified incubator at 37°C in a 5% CO_2_ atmosphere. The GSPs were dissolved in a small amount of dimethylsulfoxide (DMSO, 100 μL) prior to addition to the media. The maximum concentration of DMSO in the media did not exceed 0.1% (v/v). Cells treated with DMSO only served as a vehicle control.

### Cell viability and cell death assays

The effect of GSPs on the viability of pancreatic carcinoma cells was determined using MTT assay as described previously [24]. Briefly, cells were treated with or without GSPs for 24 and 48 h. At the end of stipulated time, cells were treated with 50 µL of 5 mg/mL MTT and the resulting formazan crystals were dissolved in 150 µL of DMSO. The color absorbance was recorded at 540 nm using a Bio-Rad 3350 microplate reader. The effect of GSPs on cell viability was calculated in terms of percent of control, which was arbitrarily assigned a value of 100% viability. GSPs-induced cell death was determined using a trypan blue dye exclusion assay as described previously [24]. Briefly, cells were treated with or without GSPs for 24 and 48 h. Thereafter, cells were harvested, treated with 0.25% trypan blue dye and the cells that had taken up the dye were counted under a microscope using a hemocytometer. The GSPs-induced cell death is expressed as the mean ± SD percentage of dead cells in each treatment group from three repeated experiments.

### Cell cycle analysis

Miapaca-2 and PANC-1 cells were treated with different concentration of GSPs (0, 20, 40 and 60 µg/mL) in complete medium for 48 h. The cells were then harvested, and processed for cell cycle analysis, as detailed previously [24]. Briefly, the 1×10^5^ cells were re-suspended in 50 µL cold PBS to which 450 µL cold methanol was added and the cells were then incubated for 1 h at 4°C. After centrifugation, the pellet was incubated with RNase A (20 µg/mL) for 30 min. The cells were incubated with propidium iodide (50 µg/mL) on ice in the dark. The cell cycle distribution of the cells was then determined using a FACS Calibur instrument (BD Biosciences, San Jose, CA) equipped with Cell Quest 3.3 software in the Core Facility of the UAB Comprehensive Cancer Center.

### Apoptotic cell death analysis by flow cytometry

GSPs-induced apoptotic cell death in pancreatic cancer cells was quantitatively determined by flow cytometry using the Annexin V-conjugated Alexa fluor 488 Apoptosis Detection Kit following the manufacturer's protocol, as previously described [24]. Briefly, after treatment of cells with GSPs for 48 h, cells were harvested, washed with PBS and incubated with Annexin V Alexa fluor488 (Alexa488) and propidium iodide for 10 min in the dark. The stained cells were then analyzed by fluorescence activated cell sorting using the FACSCalibur instrument (BD Biosciences) and Cell Quest 3.3 software.

### Western blot analysis

Following treatment of pancreatic cancer cells with or without GSPs the cells were harvested, washed with cold PBS, and lysed with ice-cold lysis buffer supplemented with protease inhibitors as detailed previously [24]. For western blot analysis, proteins were resolved on 10% Tris-glycine gels and transferred onto a nitrocellulose membrane. After blocking the non-specific binding sites, the membrane was incubated with the primary antibody at 4°C overnight. The membrane was then incubated with the appropriate horseradish peroxidase-conjugated secondary antibody and the immunoreactive protein bands were visualized using enhanced chemiluminescence reagents (Amersham Biosciences, Piscataway, NJ). The membrane was then stripped and reprobed with anti-β-actin antibody to verify equal protein loading on the gel.

### Animals and Tumor xenograft model

Female athymic nude mice of 4–5 weeks of age were purchased from the National Cancer Institute (Bethesda, MD) and housed in the Animal Resource Facility at the University of Alabama at Birmingham in accordance with the Institutional Animal Care and Use Committee guidelines. The animal protocol used in this study was approved by the Institutional Animal Care and Use Committee (IACUC) of the University of Alabama at Birmingham, and the animal protocol number is: 101109267. Mice were provided with a sterilized AIN76A diet and water *ad libitum*. To determine the *in vivo* chemotherapeutic efficacy of dietary GSPs against human pancreatic tumor xenograft growth, exponentially growing Miapaca-2 cells (5×10^6^ in 100 µL PBS) were injected subcutaneously in the right flank of each mouse. One day after tumor cell inoculation, animals were divided randomly into two groups with eight mice per group. One group of mice received the AIN76A control diet, while the second group of mice received a 0.5% GSPs-supplemented AIN76A control diet in pellet form throughout the experiment period. The experiment was terminated at the 11^th^ week after tumor cell inoculation. The tumor growth and body weight per mouse per week was recorded. Tumor size was measured using Vernier calipers and volumes were calculated using the hemiellipsoid model formula: tumor volume  = ½ (4π/3) (l/2) (w/2) h, where l  =  length, w  =  width and h  =  height. Based on the IACUC guidelines, tumor size should not increase more than 1.5 cm^2^ or size of a dime. At this stage, the experiment was terminated, mice were sacrificed, tumor from each mouse was excised and the wet weight of each tumor in each group was recorded. A portion of the tumor was used to prepare tumor lysates for western blot analysis and the other portion of the tissue was paraffin-embedded and used for immunohistochemical analysis.

### Immunohistochemical detection of Ki-67-positive, p-Akt-positive and activated caspase-3-positive cells

Paraffin-embedded tumor sections (5 µm thick) were deparaffinized and rehydrated, as described previously [25]. Following rehydration, antigen retrieval was carried out by placing the slides in 10 mmol/L sodium citrate buffer (pH 6.0) at 95°C for 20 min followed by 20-min cooling. The sections were then washed in PBS and non-specific binding sites were blocked with 1% bovine serum albumin with 2% goat serum in PBS before incubation with either anti-Ki-67, anti-p-Akt or anti-cleaved caspase-3 antibody. After washing, the sections were incubated with biotinylated secondary antibody followed by horseradish peroxidase-conjugated streptavidin. The sections were further incubated with 2,4-diaminobenzidine substrate and counterstained with hematoxylin. The Ki-67-positive and cleaved caspase 3-positive cells in a section were counted in at least 4-5 different fields and photographed using an Olympus microscope (Model BX40F4, Tokyo, Japan) fitted with a Q-color 5 Olympus camera.

### TUNEL assay for apoptotic cells

The TUNEL assay was performed using DeadEnd^TM^ Colorimetric TUNEL System Kit (Promega Corporation, USA) following the manufacturer's protocol. Briefly, after antigen retrieval, the tumor sections (5 µm-thick) were fixed by incubation with 4% paraformaldehyde at 4°C. The permeabilized sections were incubated with terminal deoxynucleotidyl transferase recombinant (rTdT) enzyme-catalysed reaction and nucleotide mixture for 60 min at 37°C in the dark. After immersion in stop/wash buffer for 15 min at room temperature, the sections were washed with PBS to remove unincorporated fluorescein-12-dUTP and the nuclei counterstained with hematoxylin. TUNEL-positive cells were examined and counted under microscope. The TUNEL-positive cells are expressed as a percentage of total cells in the microscopic field.

### Statistical analysis

The statistical significance of the difference between the values of control and treatment groups was determined by either Student *t* test or simple one-way ANOVA followed by Tukey's *post hoc* test for multiple comparisons using GraphPad Prism version 4.00 for Windows, GraphPad Software, San Diego, California, USA, www.graphpad.com. In each case, *P*<0.05 was considered statistically significant.

## Results

### GSPs inhibit viability and induce death of pancreatic cancer cells

**Figure 1:**
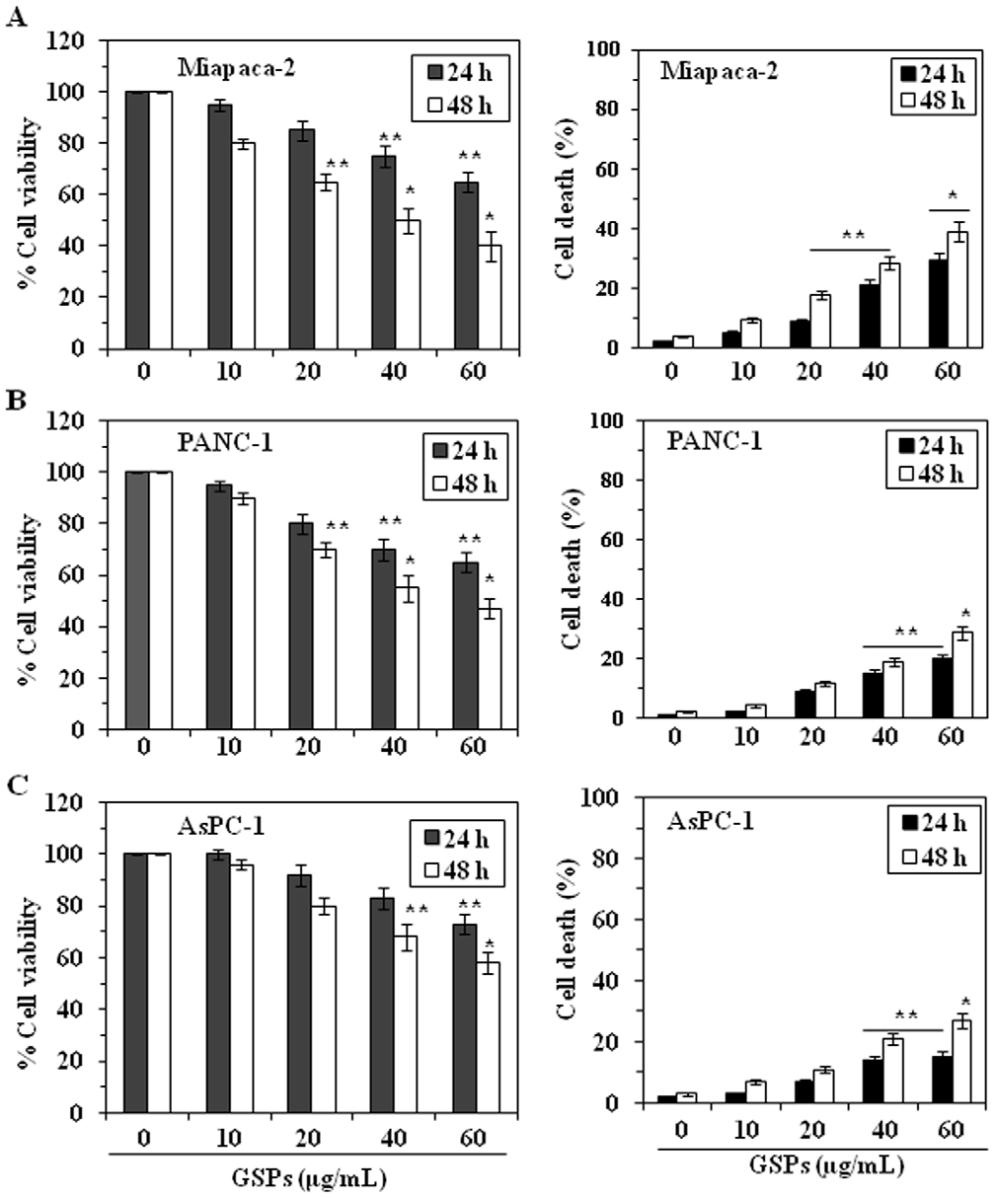
Treatment of pancreatic cancer cells with GSPs reduces their proliferation potential and induces cell death. (**A**) Miapaca-2 cells, (**B**) PANC-1 cells, and (**C**) AsPC-1 cells were treated with the indicated concentrations of GSPs for the indicated time periods. Left panels: Viability of the cells was determined using the MTT assay as described in the Materials and Methods. The data are expressed in terms of percent of control cells (non-GSPs treated) as the mean ± SD of 8 replicates. Right panels: The cytotoxic effect of GSPs on pancreatic cancer cells was determined using the trypan blue dye exclusion assay as described in Materials and methods. The cell death data are presented as the mean percent of dead cells from three experiments ± SD *vs.* control group (non-GSPs-treated). Significant difference *vs.* control group, ^*^ *P*<0.001; ^**^ *P*<0.05.

The antiproliferative effects of GSPs on pancreatic cancer cell lines, Miapaca-2, PANC-1 and AsPC-1, were determined using an MTT assay. The cells were treated with different concentrations of GSPs (0, 10, 20, 40 and 60 µg/mL) for 24 and 48 h. A dose-dependent reduction in the viability of the Miapaca-2 cells was observed that ranged from 2 to 35% (*P*<0.05) after 24 h, and 20 to 60% (*P*<0.01) after 48 h of treatment with GSPs, as shown in Figure 1A. Under identical conditions, similar effects of GSPs were observed on treatment of PANC-1 and AsPC-1 cells (Figure 1B and 1C, left panels) except that the GSPs-induced reduction of the viability of AsPC-1 cells was less than that observed for the other cell lines. We also determined the cytotoxic effect of GSPs on the pancreatic cancer cell lines in terms of cell death using the trypan blue dye exclusion assay. As shown in Figure 1A (right panels), when compared with the non-GSPs-treated control cells, treatment of Miapaca-2 cells with GSPs resulted in a significant and dose-dependent increase in cell death. Treatment for 24 h resulted in a 5–28% (*P*<0.05) increase in cell death, while treatment for 48 h resulted in 9–38% (*P*<0.05–0.001) cell death. Using identical conditions, similar cytotoxic effects were observed on treatment of PANC-1 and AsPC-1 cells with GSPs (Figure 1B and 1C, right panels).

### GSPs induce G2-M phase cell cycle arrest in pancreatic cancer cells

**Figure 2:**
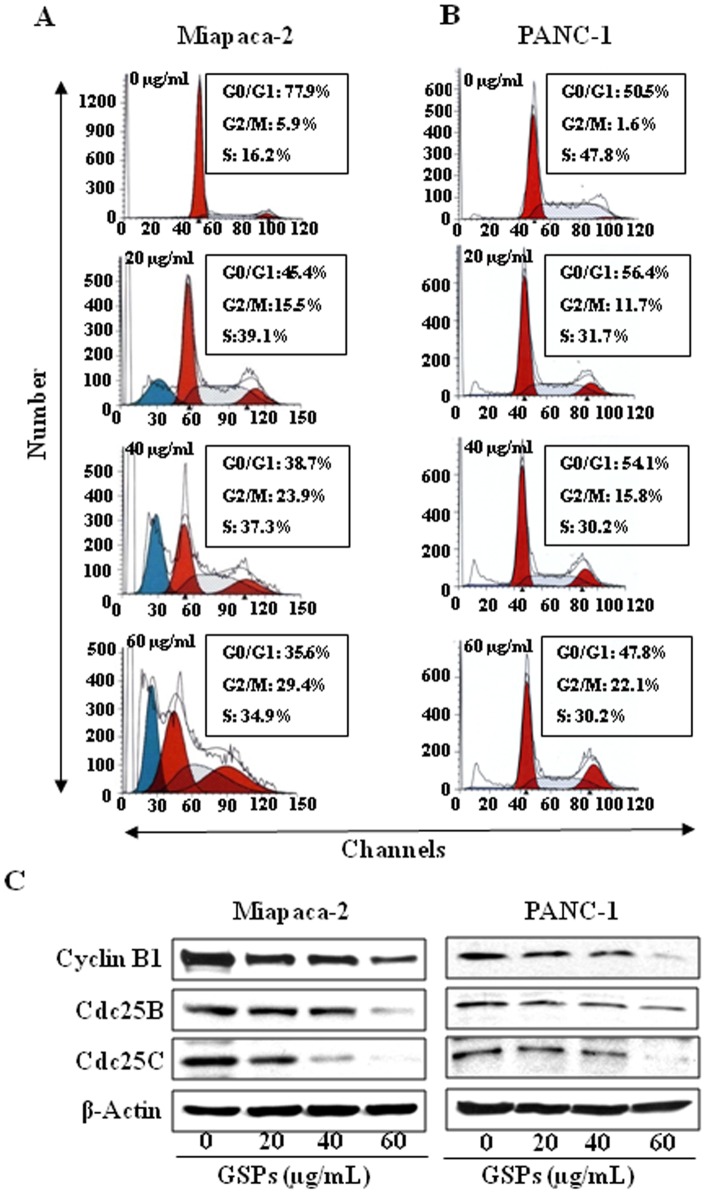
Effect of GSPs on cell cycle progression of pancreatic cancer cells. Miapaca-2 and PANC-1 cells were treated either with vehicle (0.1% DMSO) or GSPs (20, 40 and 60 µg/mL) in complete medium. After 48 h of treatment, cells were harvested and digested with RNase. Cellular DNA was stained with propidium iodide and flow cytometric analysis was performed to analyze the cell cycle distribution, as detailed in the Materials and Methods. The representative cell cycle histograms from two independent experiments in Miapaca-2 (**A**) and PANC-1 (**B**) cells after treatment with different doses of GSPs are shown. (**C**) Effect of GSPs on G2/M phase cell cycle regulatory proteins in Miapaca-2 and PANC-1 cells. The cells were treated with either vehicle or GSPs (20, 40, 60 µg/mL in DMSO) for 48 h and thereafter harvested, cell lysates prepared and then subjected to SDS-PAGE followed by western blot analyses for various proteins. β-actin was used to verify equal loading of the samples. Representative blots from three individual experiments are shown.

Disruption of the normal cell-cycle progression and division are important events in the development of cancer. To determine the possible mechanisms of the anti-proliferative effects of GSPs, cell cycle analysis was undertaken using Miapaca-2 and PANC-1 cell lines treated with 20, 40 or 60 µg/mL of GSPs. As shown in Figure 2A, after treatment of Miapaca-2 cells with GSPs for 48 h there were significantly higher number of cells in the G2-M phase at all the concentrations used: 20 µg/mL (15.5%, *P*<0.05), 40 µg/mL (23.9%, *P*<0.01) and 60 µg/mL (29.4%, *P*<0.001) as compared to the non-GSPs-treated controls (5.9%). At the 24 h time-point, the effect of GSPs on G2/M cell cycle arrest was significantly less than that observed after treatment for 48 h. Similar effects of GSPs on G2/M phase arrest were found using the PANC-1 cell line (Figure 2B). We next determined the effect of GSPs on cell cycle regulatory proteins involved in the G2/M phase of the cell cycle progression. The results of western blotting revealed that treatment of Miapaca-2 and PANC-1 cells with varying concentrations of GSPs for 48 h resulted in a dose-dependent decrease in the cyclin B1 expression as well as a reduction in the expression levels of Cdc25B and Cdc25C proteins which regulate the G2/M phase of cell cycle (Figure 2C).

### GSPs induce apoptosis of pancreatic cancer cells

**Figure 3:**
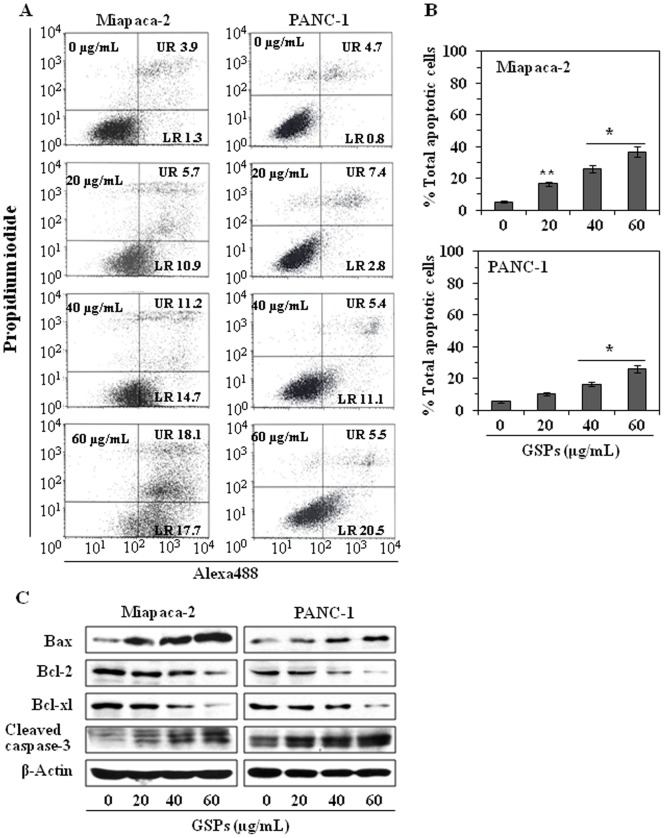
GSPs induce apoptosis in human pancreatic cancer cells in a dose-dependent manner. (**A**) Miapaca-2 and PANC-1 cells were treated with different concentrations of GSPs (0, 20, 40 and 60 µg/mL) in complete medium for 48 h, then harvested for the analysis of apoptotic cells by FACS analysis using the Annexin V-Alexa Fluor488 Apoptosis Vybrant Assay Kit (Alexa488) following the manufacturer's protocol. The lower right (LR) quadrant of the FACS histograms indicates the percentage of early apoptotic cells (Alexa488-stained cells) and the upper right (UR) quadrant indicates the percentage of late apoptotic cells (Alexa488+ propidium iodide-stained cells). (**B**) Total percentages of apoptotic cells in each treatment group after 48 h are summarized with data presented as the mean ± SD of two experiments. Significant difference *vs.* control group (non-GSPs-treated), *P<0.001; **P<0.05. (**C**) Treatment of Miapaca-2 and PANC-1 cells with varying concentrations of GSPs for 48 h results in a dose-dependent reduction in the expression levels of the anti-apoptotic proteins (Bcl-2 and Bcl-xl) while increasing the expression of the pro-apoptotic protein Bax. GSPs also increase the activation level of caspase-3 in cells.

To examine whether the reduction in cell viability and induction of G2/M phase arrest in human pancreatic cancer cells by GSPs treatment was related to the induction of apoptosis, the Miapaca-2 and PANC-1 cells were treated with varying concentrations of GSPs and the percentage of apoptotic cells were assessed using the Alexa488 Apoptotic Detection Kit. Apoptotic cell death was determined in terms of early- or late-stage apoptotic cells, which are shown respectively in the lower right (LR) and upper right (UR) quadrants of the FACS histograms (Figure 3A). Treatment of the Miapaca-2 and PANC-1 cells with GSPs for 48 h resulted in significant induction of apoptosis in both cell lines. The percentages of total apoptotic cells (in UR+LR quadrants) in Miapaca-2 cells after GSPs treatments were as follows: 5.2% (vehicle-treated control), 16.6% (20 μg/mL, *P*<0.05), 25.9% (40 µg/mL, *P*<0.01), and 35.8% (60 µg/mL, *P*<0.001), as summarized in Panel B. Similar GSPs-induction of apoptosis was observed when PANC-1 cells were treated with GSPs for 48 h, as shown in Figure 3A and 3B.

### Effect of GSPs on the proteins of Bcl-2 family in pancreatic cancer cells

Members of the Bcl-2 family of proteins play crucial roles in regulation of apoptosis by functioning as promoters or inhibitors of cell death [26]–[28]. Western blot analysis revealed that treatment of Miapaca-2 cells with GSPs (20, 40, 60 µg/mL) for 48 h resulted in a dose-dependent reduction in Bcl-2 and Bcl-xl protein expression (Figure 3C) whereas the expression of Bax was markedly up-regulated with increasing concentrations of GSPs (Figure 3C). Similar effect of GSPs on the proteins of Bcl-2 family was also found when PANC-1 cells were treated with GSPs for 48 h. As cleaved caspase 3 is considered to be a hallmark of apoptosis, we also determined the effect of GSPs on the cleaved caspase-3 in these samples and confirmed that the GSPs increased the activation or cleavage of caspase-3 in both Miapaca-2 and PANC-1 cells (Figure 3C).

### GSPs inhibit PI3K/Akt expression in cancer cells

**Figure 4:**
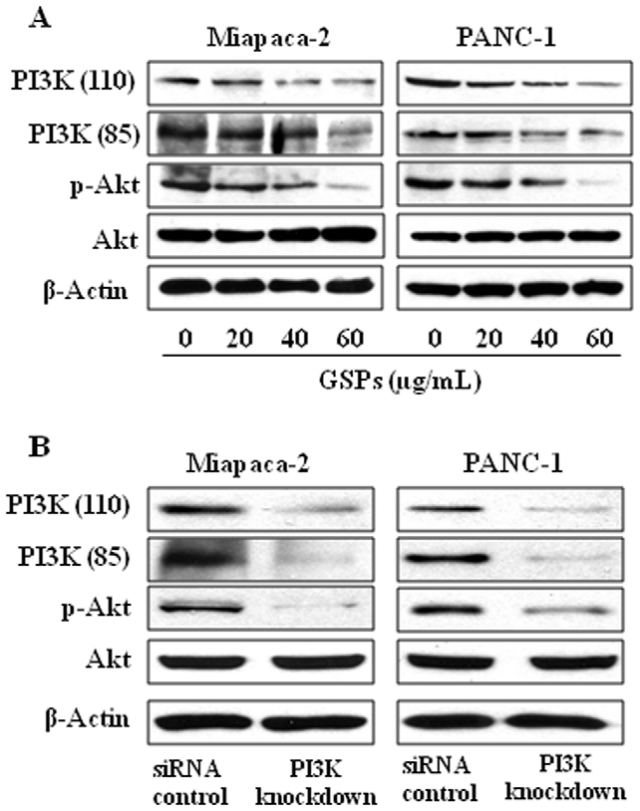
Effect of GSPs on PI3K signaling proteins in pancreatic cancer cells. (**A**) Treatment of Miapaca-2 and PANC-1 cells with GSPs decreases the expression level of PI3Kp85 (regulatory) and PI3Kp110 (catalytic), and phosphorylation of Akt in a dose-dependent manner. After treatment for 48 h, cells were harvested, and cell lysates were subjected to western blot analysis to determine the expression of different proteins. β-actin was used to verify equal loading of the protein samples. (**B**) siRNA knockdown of PI3K in pancreatic cancer cells resulted in reduction of p-Akt levels compared to siRNA control group.

As the PI3K/Akt signaling pathway plays a critical role in cancer cell survival and development of cancers, we examined the effects of GSPs on this cell survival pathway. Miapaca-2 and PANC-1 cells were treated with GSPs for 48 h, cell lysates prepared and the levels of PI3K and phosphorylation of Akt at Ser^473^ determined by western blot analysis. The results revealed that treatment of these cell lines with GSPs for 48 h decreased the levels of both the regulatory (p85) and catalytic (p110) subunits of PI3K and also reduced the phosphorylation of Akt at Ser^473^ in a concentration-dependent manner (Figure 4A). As it is well known that Akt activity is usually regulated by activated PI3K, we further verified that Akt is regulated by PI3K in pancreatic cancer cells. For this purpose, we knocked-down PI3K in Miapaca-2 and PANC-1 cells using PI3K siRNA kit (Santa Cruz Biotechnology, Inc.) following the manufacturer's protocol. As shown in Figure 4B, western blot analysis revealed that siRNA knockdown of PI3K resulted in marked reduction in the levels of p-Akt in both Miapaca-2 and PANC-1 cells. However, the effect on total Akt expression was not observed in these cell lines on siRNA knockdown of PI3K. Knockdown of PI3K proteins was also verified using western blotting.

### Dietary GSPs inhibit the growth of pancreatic tumor xenografts in athymic nude mice

As these *in vitro* studies indicated that treatment of pancreatic cancer cells with GSPs reduces the viability and induces apoptosis of these cells, we sought to determine whether dietary administration of GSPs inhibits *in vivo* tumor growth using a xenograft mouse model. As the therapeutic effect of GSPs on Miapaca-2 and PANC-1 cell lines were identical, we selected the Miapaca-2 cell line for these *in vivo* studies. In previous studies we also have found that dietary GSPs at the dose of 0.5% induced significant growth inhibitory effects on tumors [9], [29], we selected this dose of GSPs for further *in vivo* study. The mice were given the AIN76A control diet alone or the same diet supplemented with GSPs (0.5%, w/w). The average body weights of the GSPs-treated and non-GSPs-treated mice were comparable throughout the experimental period (data not shown). Similarly, the mice that were given GSPs in their diet did not exhibit any physical sign of toxicity or abnormal behavior (data not shown). These data suggest that administration of GSPs in the diet at the concentration used in these studies is not associated with apparent gross toxicity.

**Figure 5:**
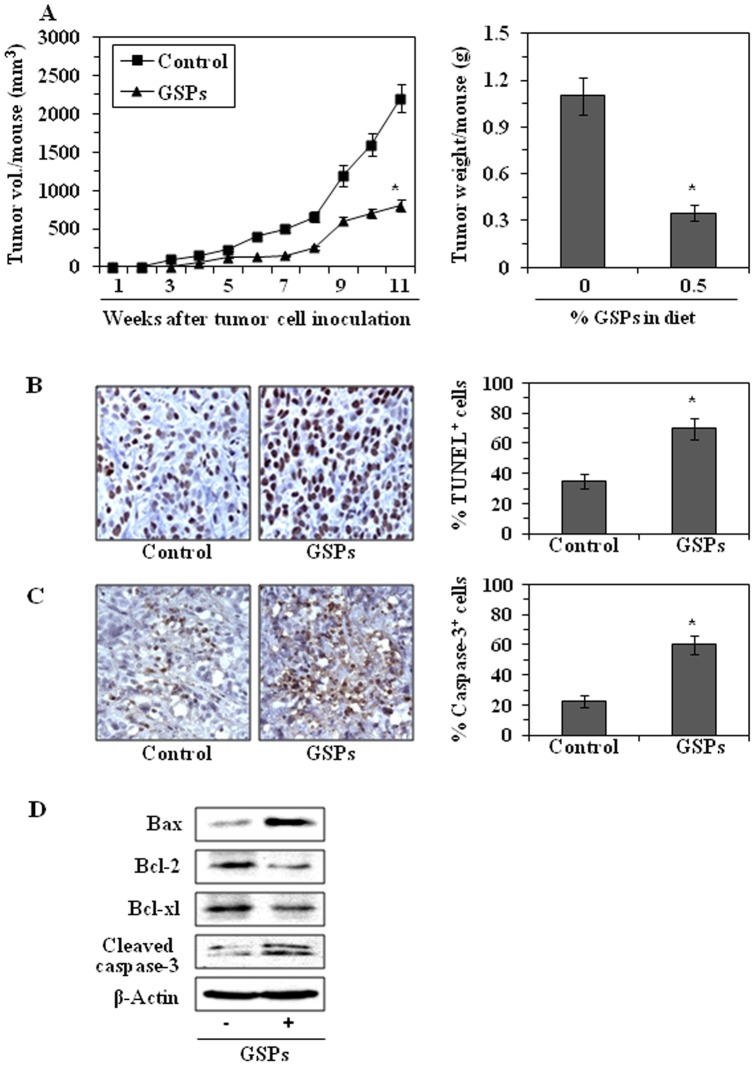
Dietary GSPs inhibit *in vivo* tumor xenograft growth in athymic nude mice. (**A**) Dietary administration of GSPs (0.5%, w/w) inhibits the growth of Miapaca-2 cells grown as xenografts in athymic nude mice. Average tumor volume ± SD/mouse (mm3) in each treatment group was measured on weekly basis. Tumor xenograft tissues were harvested at the termination of the experiment at 11 weeks and the wet weight of the tumor/mouse in each group is reported in grams as the mean ± SD. Statistical significance of difference between control and GSPs-treated groups was analyzed by one-way ANOVA followed by the Bonferroni *t* test, n = 8. Statistical significance *vs* tumors from non-GSPs-treated control mice, *P<0.005. (**B**) The immunohistochemical detection of TUNEL-positive cells in tumor xenograft tissues from mice administered GSPs in the diet and mice not receiving GSPs are shown, and resultant data on TUNEL-positive cells are summarized. (**C**) Immunohistochemical detection of activated caspase-3-positive cells in tumor xenograft tissues from GSPs-treated and non-GSPs-treated mice. Immunohistochemical data in terms of percentage of positive cells are presented as the mean ± SD of 5–6 tumor samples from each group. TUNEL-positive or caspase-3-positive cells were counted in four to five different fields, and data are summarized in terms of percent positive cells from 5–6 tumor xenograft samples. Representative micrographs are shown. (**D**) Dietary GSPs inhibit the expression of anti-apoptotic proteins while increasing the expression of pro-apoptotic protein, Bax, and the levels of activated caspases-3 in tumor xenograft tissues. Tumor lysates were prepared from the tumor xenografts collected at the termination of the experiment described in Figure 5A and subjected to western blot analysis, as described in Materials and methods. Representative blots are shown from independent experiments from at least five tumors from five different mice per group with identical observations.

Weekly measurement of the tumor volume indicated that the average tumor growth in terms of total tumor volume/mouse was higher in the non-GSPs-treated mice than the GSPs-treated groups. As shown in Figure 5A, in mice that received GSPs at the dose of 0.5% in their diet, the tumor volume at the termination of the experiment was 64% (*P*<0.005) less. The experiment was terminated on the 11^th^ week after tumor cell implantation. At this time the mice were sacrificed, the tumors harvested, and the wet weight of the tumor/mouse in each treatment group was recorded. As shown in Figure 5A (right panel), the wet weight of the tumors was 68% lower (*P*<0.005) in mice administered 0.5% GSPs in the diet as compared to the tumors from non-GSPs-treated mice.

### Dietary GSPs enhance apoptotic cell death of pancreatic tumor cells in xenografts

To determine whether inhibition of tumor growth by dietary GSPs is caused by the death of tumor cells in xenograft tissues, we evaluated the effect of GSPs on the apoptotic index of tumor cells in xenograft samples using a TUNEL assay. Immunohistochemical analysis revealed greater numbers of TUNEL-positive cells in the samples of xenografts from the group of mice administered GSPs (0.5%, w/w) as compared with the numbers in the samples of xenografts from the mice that did not receive GSPs (Figure 5B). The numbers of TUNEL-positive cells in the GSPs-treated and non-GSPs-treated tumors were counted and a summary of these data is provided in Figure 5B (right panel). Immunohistochemical detection of activated caspase-3-positive cells in tumor xenograft samples revealed that the numbers of caspase-3-posive cells were higher in tumors from GSPs-fed mice than in the tumors of non-GSPs-fed mice (Figure 5C). The percentage of activated caspase-3-positive cells in tumor samples from mice administered GSPs was higher (60%) than the percentage of caspase-3-positive cells in the tumors of mice that did not receive GSPs (23%). Western blot analysis of cleaved caspase-3 in lysates of tumor cells confirmed that GSPs increased the levels of cleaved caspase-3 (Figure 5D). In parallel, western blot analysis of the expression of Bcl-2 family proteins in the xenograft tumor lysates revealed that dietary GSPs enhanced the levels of Bax while reducing the levels of Bcl-2 and Bcl-xl.

### Dietary GSPs inhibit the proliferative potential of tumor cells and inhibit the PI3K/Akt pathway

**Figure 6:**
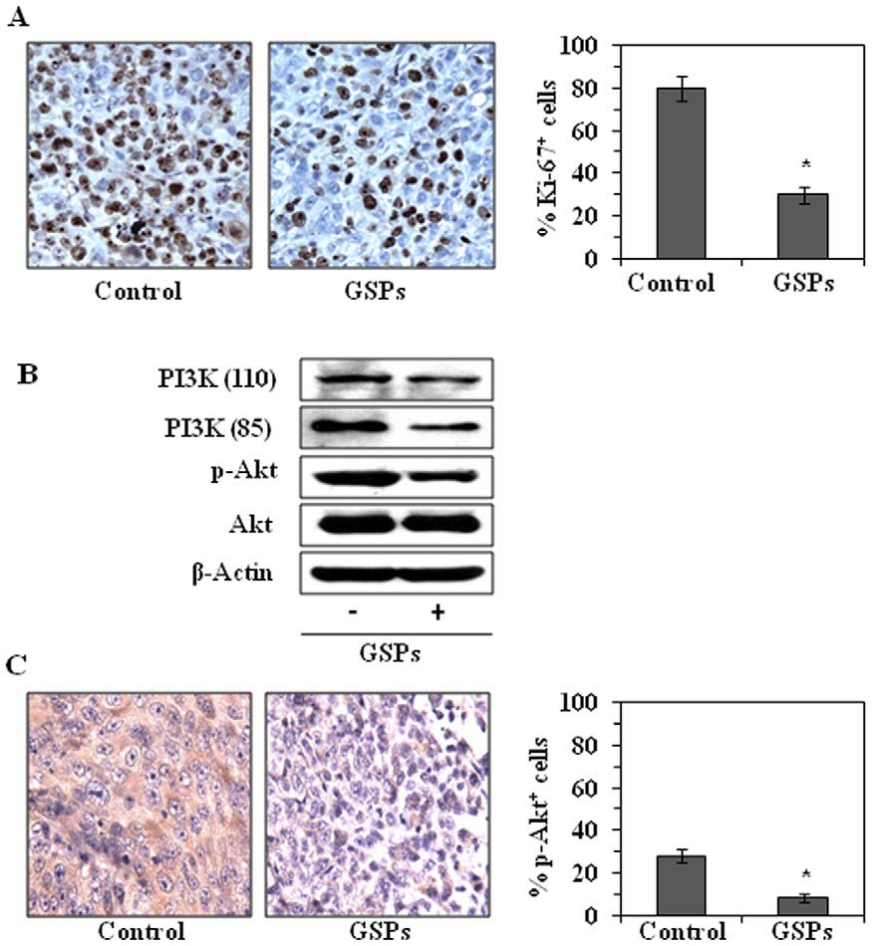
Dietary GSPs reduce the proliferation potential and inhibit PI3K signaling pathway in xenograft tissues. (**A**) Immunohistochemical analysis of tumor samples obtained from mice at the termination of the experiment described in Figure 5 was used to detect Ki-67-positive cells as an estimate of the proliferation index. Ki-67-positive cells were counted in four to five different fields of a section, and data are summarized in terms of percent positive cells from 5 tumor xenograft samples. (**B**) Dietary GSPs reduce the levels of PI3K (p110, p85) and p-Akt in tumor xenograft tissues. Western blot analysis was performed to analyze the levels of these proteins. (**C**) Dietary GSPs decrease the levels of p-Akt in tumor samples. Immunohistochemical data show a reduction in the immunostaining intensity of p-Akt in tumor xenograft samples from GSPs-treated mice. Representative micrographs are shown from 4 tumor samples/group. Phospho-Akt-positive cells were counted in four to five different fields of a section, and data are summarized in terms of percent positive cells from 5 tumor xenograft samples (right panel). Statistical significance *vs*. tumor samples from mice receiving the control diet only, ^*^ *P*<0.001.

Uncontrolled tumor cell proliferation is a characteristic feature of most cancers. We therefore analyzed the pancreatic tumor xenografts for the potential antiproliferative effects of GSPs using immunohistochemical detection of Ki-67-positive cells. For this purpose, tumor xenograft samples from mice administered GSPs (0.5%, w/w) in the diet and from mice that received the control diet as described above, were used. The results of the immunohistochemical detection of Ki-67-positive cells in tumor xenograft tissues (Figure 6A) indicated that the percentage of proliferating cells was significantly lower (40%, *P*<0.01) in tumor xenografts from GSPs-treated mice than in the tumor xenografts from the control mice (Figure 6A, right panel).

Next, we determined the effect of GSPs on PI3K/Akt pathway in tumor xenograft samples. As shown in Figure 6B, dietary GSPs resulted in decreased expression of PI3K (p110 and p85) proteins and reduced the phosphorylation of Akt in the tumor xenografts. Immunohistochemical analysis further suggested that the staining intensity of p-Akt was lower in xenograft tumor samples from the mice administered dietary GSPs as compared to the intensity of staining of tumor xenograft samples from the control mice that did not receive GSPs (Figure 6C, left panel). The results of the immunohistochemical analysis of p-Akt-positive cells in tumor xenograft tissues revealed that the percentage of p-Akt-positive cells were significantly lower (*P*<0.01) in tumor xenografts from GSPs-treated mice than in the tumor xenografts from the control mice (Figure 6C, right panel).

## Discussion

Bioactive components from plant origin offer promising options for the development of effective strategies for the prevention or treatment of cancers. GSPs are promising bioactive molecules that have demonstrated http://carcin.oxfordjournals.org/cgi/content/full/24/8/1379 – B10#B10anti-carcinogenic effects in some animal tumor models. Currently, there is no effective treatment of pancreatic cancer as conventional chemotherapy and radiation therapy have shown only limited success in improving patient survival. We therefore undertook a comprehensive analysis of the effects of GSPs on pancreatic cancer cells using both *in vitro* and *in vivo* models. Here, we report that GSPs significantly reduced the viability and induce cell death/apoptosis of Miapaca-2, PANC-1 and AsPC-1 human pancreatic cancer cell lines, suggesting that GSPs may have a beneficial therapeutic effect in pancreatic cancer.

It has been recognized that control of cell cycle progression in cancer cells is an effective strategy to inhibit tumor growth [30], [31] as the molecular analyses of human cancers revealed that cell cycle regulators are frequently deregulated in most of the common malignancies [32], [33]. Our *in vitro* data demonstrate that treatment of Miapaca-2 and PANC-1 cells with GSPs induces G2/M phase arrest of cell cycle progression indicating that one of the mechanisms by which GSPs reduce the viability of pancreatic cancer cells is inhibition of cell cycle progression. The growth arrest of cancer cells in G2/M phase provides an opportunity for cells to either undergo repair mechanisms or undergo apoptosis. In most advanced malignancies, the cancer cells become resistant to apoptosis and/or do not respond to the cytotoxic effects of chemotherapeutic agents [33]. We therefore determined the effect of GSPs on the induction of apoptosis in Miapaca-2 and PANC-1 cells. FACS analysis indicated that treatment of both cell lines with GSPs resulted in a significant induction of apoptosis. Induction of apoptosis in cancer cells is thought to act as a protective mechanism against neoplastic development by eliminating those cells which have been improperly stimulated for proliferation [34]. Acquired resistance towards most endogenous apoptosis signaling mechanisms is considered as a hallmark of most of the cancers. Therefore, GSPs appears to be a potent chemotherapeutic agent against pancreatic cancer cells. Usually, the induction of apoptosis in cancer cells involves the up-regulation of pro-apoptotic proteins and/or down-regulation of anti-apoptotic proteins. A major apoptotic signal transduction cascade associated with induction of apoptosis includes Bcl-2 family members [35], which either promote cell survival or promote apoptosis [35], [36]. We found that treatment of Miapaca-2 and PANC-1 cells with GSPs resulted in a dose-dependent decrease in the levels of anti-apoptotic proteins and a simultaneous increase in the pro-apoptotic protein Bax. These effects of GSPs lead to the activation of caspase-3 in cells, which is a marker of apoptosis. These data suggest that GSPs-induced apoptosis in pancreatic cancer cells is mediated through alterations in the levels of proteins of Bcl-2 family and the activation of caspases-3, and this may be a possible mechanism of GSPs-induced apoptosis in pancreatic cancer cells.

PI3K/Akt is a potent survival pathway that has been implicated in the acquired resistance to apoptotic cell death associated with treatment with chemotherapeutic drugs and radiation therapy in a variety of cancers including pancreatic cancer [22], [23]. Our study clearly demonstrates that treatment of Miapaca-2 and PANC-1 cell lines with GSPs decreases the expression of both regulatory (p85) and catalytic (p110) subunits of PI3K and phosphorylation of Akt at Ser^473^. Thus it can be suggested that inhibition of cell proliferation by GSPs is mediated, at least in part, through the down-regulation of PI3K/Akt pathway.

Although *in vitro* cell culture models are a good system for preliminary screening of the effects of chemotherapeutic agents; the observations must be verified *in vivo* using animal models prior to their potential consideration of their use in humans. We therefore used an *in vivo* model of xenografts of Miapaca-2 tumor cells in athymic nude mice to verify the chemotherapeutic potential of GSPs against pancreatic tumor cell growth. The GSPs were administered in the diet of the mice as this approach has proven effective in other cancers [9], [29]. Our study provides evidence that dietary administration of GSPs inhibits the growth of Miapaca-2 pancreatic tumor xenografts without any apparent sign of toxicity in the athymic nude mice. The identification of biomarkers is an important consideration in terms of monitoring the efficacy of cancer chemopreventive or therapeutic strategies, in suggesting potential combinations with other agents, and in elucidating the mechanisms of action of any test agent. In this context, the inhibition of the growth of tumor xenograft in athymic nude mice by dietary GSPs was also associated with the induction of apoptotic cell death of tumor cells, as indicated by the analysis of TUNEL-positive and activated caspase-3-positive cells in tumor xenograft samples. GSPs also modulated the levels of proteins of Bcl-2 family in favor of tumor cell apoptosis and decreased *in vivo* tumor cell proliferation, at least in part, by targeting PI3K/Akt cell survival pathway.

In summary, the results of this study show for the first time the chemotherapeutic efficacy of GSPs on the growth of human pancreatic cancer cells *in vitro* and tumor xenograft growth *in vivo*. Thus GSPs appear to be an attractive dietary bioactive phytochemical for pancreatic cancer chemoprevention and/or treatment.
